# Grand Challenges in Community Oral Health

**DOI:** 10.3389/froh.2020.00004

**Published:** 2020-07-14

**Authors:** Fawad Javed

**Affiliations:** Division of Orthodontics and Dentofacial Orthopedics, Eastman Institute for Oral Health, University of Rochester, Rochester, NY, United States

**Keywords:** Community Oral Health, oral diseases, epidemiology, questionnaire, health education, health policy

Community Oral Health is usually considered a sub-category of dentistry; however, it intersects three domains, namely (a) dentistry; (b) medicine; and public health [1]. The multifaceted interaction among oral health, systemic diseases, and health outcomes impedes forthright clarifications as to their relationships. There is mounting acknowledgment by oral healthcare providers of the need to assess the general health status of patients, which may otherwise jeopardize oral health or complicate treatment/s [2]. Besides traditional risk-factors such as habitual use of tobacco products and systemic conditions such as obesity; [3, 4] an underprivileged socio-economic and literacy status are additional factors that are linked with a compromised oral and systemic health of patients [3]. Social inequalities in health and health care are usually manifested in the oral cavity, even as they are intimately associated with other systemic diseases [5]. Access to oral and dental care is mandatory for all communities as socioeconomically underprivileged, ethnic/racial minorities and underserved populations tackle obstacles, which require ingenuity and flexibility to overcome.

Patient education through community-based health promotion and education programs are essential to elucidate the significance oral health toward improvement in the overall quality of life (QoL). For instance, individuals habitually using tobacco products such as cigarettes, pipe, *bidis*, and cigars, often perceive that nicotine consumption using waterpipe (shisha or narghile or hookah) or electronic nicotine delivery systems (ENDS) (such as electronic-cigarettes) is less deleterious to health than conventional smoking. However, scientific data has confirmed that use of waterpipe and ENDS is not a safe alternate to traditional cigarette-smoking [6–8]. Likewise, it is known that systemic conditions that suppress the immune system (such as poorly-controlled diabetes mellitus, and acquired immune deficiency syndrome) are known to jeopardize oral health [3, 9]. It is essential to formulate and implement policies/programs related to health education/promotion to educate the community about the bidirectional connection between oral and systemic health. Moreover, such policies/programs may help enhance awareness in the community about the benefits of oral and general health maintenances toward improvement of QoL; and simultaneously elucidate the consequences of a compromised oral and systemic health on the overall QoL. In this regard, epidemiological studies based on patient's perspective and community-based efforts to educate the community about the deleterious effects of nicotinic products are welcomed; and Community Oral Health section of the journal *Frontiers in Oral Health* is an excellent platform to highlight this aspect of oral health and related research.

The Community Oral Health section of the journal *Frontiers in Oral Health* is developed to serve as a platform that elucidates and focuses on the scientific-based information related to oral health education and promotion in the global community. The intention is to constantly expand the knowledge base in the field of community health. This section encourages manuscripts that display methodologically-detailed scientific findings based on original data or analysis of existing data sources; and studies with new findings will be particularly favored. Confirmatory studies based on previously known findings may also be of interest; however, the Community Oral Health section pursues to avoid repetition of known facts. This section invites original articles, review articles, commentaries, Letters to the Editors, and perspectives on interventions related to prevention of oral diseases (including but not limited to dental caries, fluorosis, periodontal diseases, peri-implant diseases, oral premalignant, and malignant lesions and tobacco-induced diseases) in a community-based setting. Questionnaire-based studies, and studies related to patients' perception and motivation toward the prevention and treatment of oral inflammatory conditions are of interest for our journal. Moreover, original and review studies focusing on oral health promotion/education in community-based dwellings are of particular interest to the readership of the Community Oral Health section of the journal *Frontiers in Oral Health*.

## Author Contributions

The author confirms being the sole contributor of this work and has approved it for publication.

## Conflict of Interest

The author declares that the research was conducted in the absence of any commercial or financial relationships that could be construed as a potential conflict of interest.
